# Identification of lncRNA‐associated differential subnetworks in oesophageal squamous cell carcinoma by differential co‐expression analysis

**DOI:** 10.1111/jcmm.15159

**Published:** 2020-03-12

**Authors:** Wei Liu, Cai‐Yan Gan, Wei Wang, Lian‐Di Liao, Chun‐Quan Li, Li‐Yan Xu, En‐Min Li

**Affiliations:** ^1^ The Key Laboratory of Molecular Biology for High Cancer Incidence Coastal Chaoshan Area Shantou University Medical College Shantou China; ^2^ Department of Biochemistry and Molecular Biology Shantou University Medical College Shantou China; ^3^ Department of Mathematics Heilongjiang Institute of Technology Harbin China; ^4^ Institute of Oncologic Pathology Shantou University Medical College Shantou China; ^5^ Department of Medical Informatics Harbin Medical University‐Daqing Daqing China

**Keywords:** differential co‐expression, differential subnetwork, lncRNA, oesophageal squamous cell carcinoma

## Abstract

Differential expression analysis has led to the identification of important biomarkers in oesophageal squamous cell carcinoma (ESCC). Despite enormous contributions, it has not harnessed the full potential of gene expression data, such as interactions among genes. Differential co‐expression analysis has emerged as an effective tool that complements differential expression analysis to provide better insight of dysregulated mechanisms and indicate key driver genes. Here, we analysed the differential co‐expression of lncRNAs and protein‐coding genes (PCGs) between normal oesophageal tissue and ESCC tissues, and constructed a lncRNA‐PCG differential co‐expression network (DCN). DCN was characterized as a scale‐free, small‐world network with modular organization. Focusing on lncRNAs, a total of 107 differential lncRNA‐PCG subnetworks were identified from the DCN by integrating both differential expression and differential co‐expression. These differential subnetworks provide a valuable source for revealing lncRNA functions and the associated dysfunctional regulatory networks in ESCC. Their consistent discrimination suggests that they may have important roles in ESCC and could serve as robust subnetwork biomarkers. In addition, two tumour suppressor genes (*AL121899.1* and *ELMO2*), identified in the core modules, were validated by functional experiments. The proposed method can be easily used to investigate differential subnetworks of other molecules in other cancers.

## 1 INTRODUCTION

Oesophageal carcinoma is the eighth most common and the sixth most lethal cancer with a poor 5‐year overall survival ranging from 15% to 25%.^1^ Oesophageal squamous cell carcinoma (ESCC) is the predominant histological type of oesophageal carcinoma worldwide.^2^ At present, the regulatory mechanisms underlying ESCC remain largely unknown. Based on the accumulating transcriptome data, traditional differential expression analysis has successfully identified a handful of oncogenes and tumour suppressors, including protein‐coding genes (PCGs) and miRNAs, such as *PTK6*,^3^ *Rab25*,^4^ *miR‐25* 5 and *miR‐29c*,^6^ and recently encompassed long non‐coding RNAs (lncRNAs), such as *HOTAIR*,^7^, ^8^ *AFAP1‐AS1* 9 and *lncRNA625*,^10^ due to improvement of sequencing techniques. These dysregulated genes serve as potential diagnostic or prognostic biomarkers and valuable targets for further study to understand pathologic mechanisms in ESCC.^3^, ^4^, ^5^, ^6^, ^7^, ^8^, ^9^, ^10^

Despite the enormous contributions to the field, differential expression analysis has not captured the full potential of transcriptome data. Some genetic mutations and post‐translational modifications, such as methylation, phosphorylation and acylation, can modify protein activity without affecting the gene expression level, but can alter the interaction pattern with other genes.^11^, ^12^ A well‐known example is APC, the most common mutated gene in colorectal cancer, whose frequent mutation leads to a truncated protein that lacks the binding sites for certain interacting proteins.^13^ Thus, an analysis based solely on differential expression analysis may miss some key driver genes. On the other hand, differential expression analysis treats genes individually, but does not account for the interactions among them, and it is widely accepted that understanding the mechanisms underlying disease must consider the contributions of alterations in gene interaction.^11^ Recently, differential co‐expression analysis has emerged as an effective tool that complements differential expression analysis to provide better insights of dysregulated mechanisms and indicate key driver genes.^11^, ^14^, ^15^, ^16^, ^17^, ^18^, ^19^ Differential co‐expression measures the correlation difference of a gene pair between two conditions (eg healthy and diseased samples). As co‐expressed gene pairs are more likely to have putative interactions, dependencies or coordinated activities in a given biological state, changes in co‐expression patterns between two conditions may reveal disease‐associated dysregulated mechanisms and indicate key driver genes.^19^ For human cancer, the gene co‐expression relationships in normal samples are extensively lost in matched tumour samples, such as breast cancer, colorectal cancer, lung cancer and gastric cancer.^12^, ^14^, ^20^, ^21^ Many studies have focused on this discrepancy to identify genes or gene modules that are dysfunctional in tumour samples by differential co‐expression analysis. For example, Anglani et al showed that differential co‐expression analysis was complementary to differential expression to unveil novel candidate cancer genes and improve the classic pathway enrichment analysis.^12^ In clear cell renal cell carcinoma, an *HNF4A*‐associated module was found to be functional in normal tissues but disrupted in tumour tissues, which could promote cell proliferation.^20^ Furthermore, differential co‐expression analysis has been successfully used to identify differentially co‐expressed modules, a group of genes significantly correlated under one condition but not the other, which may reflect dynamic changes in gene interaction networks.^11^, ^17^, ^18^, ^19^, ^20^, ^21^ However, for ESCC, the differential co‐expression patterns of genes have not been investigated. This promoted us to apply differential co‐expression analysis on ESCC and identify differentially co‐expressed modules, which may help to reveal the dysfunctional regulatory networks underlying ESCC development and suggest novel driver genes.

On the other hand, lncRNAs are attracting more and more attention with their widespread roles in cancer, including ESCC.^7^, ^8^, ^9^, ^10^, ^22^, ^23^ However, the function of the vast majority of lncRNAs remains enigmatic. Meaningful understanding of lncRNA function can only be achieved from detailed study on a case‐by‐case basis, which lacks candidate targets. To advance the understanding of lncRNA‐associated dysregulated mechanisms in ESCC and provide potential targets, large scale identification of differentially co‐expressed lncRNA‐PCG modules is urgently required.

Here, we construct a differential co‐expression network (DCN) based on ESCC expression data and propose a novel algorithm to identify lncRNA‐associated differential subnetworks on a large scale by integrating both differential expression and differential co‐expression. The identified differential lncRNA‐PCG subnetworks provide a valuable source for revealing lncRNA functions and the associated dysfunctional regulatory networks in ESCC. The functions of two tumour suppressor genes (*AL121899.1* and *ELMO2*), identified in the core modules, were further validated using functional experiments.

## 2 MATERIALS AND METHODS

### 2.1 Data sets

Three independent ESCC data sets were collected. The first two data sets (http://www.ncbi.nlm.nih.gov/geo/query/acc.cgi?acc=GSE53624 and http://www.ncbi.nlm.nih.gov/geo/query/acc.cgi?acc=GSE53622) were obtained from the Gene Expression Omnibus (http://www.ncbi.nlm.nih.gov/geo/). Expression profiles of 238 samples (119 paired ESCC and adjacent normal tissues) in http://www.ncbi.nlm.nih.gov/geo/query/acc.cgi?acc=GSE53624 were profiled by using the Agilent‐038314 human lncRNA + mRNA microarray V2.0 platform. We reannotated probe sets of this platform with three steps.

1. Probe sequences of the Agilent‐038314 array were aligned to PCG and lncRNA transcripts obtained from GENCODE database (GRCh38, release 21)^24^ by using BLASTn.^25^ Only probes perfectly mapped to lncRNAs or PCGs were retained.
2. Probes that mapped to both PCGs and lncRNAs were removed.
3. Probes targeting more than one PCG or lncRNA were removed.

The retained probes mapped uniquely to a PCG or a lncRNA transcript with no mismatch, resulting in 17 434 PCGs and 6252 lncRNAs. The 119 paired tissues were randomly split into a training set (60 paired tissues, ESCC‐train) and a test set (59 paired tissues, ESCC‐test). The expression profiles of 120 samples (60 paired ESCC and adjacent normal tissues, ESCC‐valid) in http://www.ncbi.nlm.nih.gov/geo/query/acc.cgi?acc=GSE53622 were processed in the same way.

The third data set contains the RNA‐sequencing data of 30 samples (15 paired ESCC and adjacent normal tissues) reported in our previous study.^10^ We extracted RPKM expression profiles by using TopHat version 2.0.6^26^ and easyRNAseq version 1.6.0.^27^ Transcripts that were not detected in more than 20% of the samples were removed, resulting in 16 178 PCGs and 3498 lncRNAs. The data were quantile normalized and log2‐transformed.

### 2.2 Construction of normal and tumour co‐expression networks

The normal co‐expression network (NCN) was constructed based on expression profiles of the 60 normal tissues in the ESCC‐train data set. For each pair of genes *i* and *j*, the Pearson correlation coefficient (PCC) $r_{\mathrm{ij}}^{N}$ and the associated *P*‐value $P_{\mathrm{ij}}^{N}$ (two‐sided Student's *t* test, Benjamini and Hochberg (BH) correction) were calculated by using the WGCNA^17^ R package. Gene pairs with $P_{\mathrm{ij}}^{N}<t_{N}$ were used to construct the NCN, where *t_N_* was the normal PCC *P*‐value threshold. The tumour co‐expression network (TCN) was constructed, using the same method as the NCN, based on expression profiles of the 60 ESCC tissues in the ESCC‐train data set. Gene pairs with $P_{\mathrm{ij}}^{T}<t_{T}$ were used to construct the TCN, where $P_{\mathrm{ij}}^{T}$ was the BH corrected PCC *P*‐value in tumour samples, and *t_T_* was the tumour PCC *P*‐value threshold.

### 2.3 Construction of the differential co‐expression network (DCN)

To test whether the difference between the PCC of a gene pair in normal samples and that in tumour samples was significant, the PCCs were transformed into *z* scores by using the Fisher transformation:

(1) $$z=\frac{1}{2}\log\left(\frac{1+r}{1-r}\right)$$

where *r* is the PCC, and *z* is the Fisher‐transformed PCC. Then, *z* is approximately normally distributed with variance $\sigma^{2}=1/\sqrt{n-3}$, where *n* is the number of the samples.^20^ The PCCs $r_{\mathrm{ij}}^{N}$ and $r_{\mathrm{ij}}^{T}$ of a gene pair (*i*, *j*) in the normal and tumour samples are transformed into $z_{\mathrm{ij}}^{N}$ and $z_{\mathrm{ij}}^{T}$ by using Equation (1), respectively. The difference between $z_{\mathrm{ij}}^{N}$ and $z_{\mathrm{ij}}^{T}$ can be measured by the following equation:

(2) $$\Delta z_{\mathrm{ij}}=\frac{z_{\mathrm{ij}}^{T}-z_{\mathrm{ij}}^{N}}{\sqrt{\frac{1}{n_{T}-3}+\frac{1}{n_{N}-3}}}$$

where *n_T_* and *n_N_* are the number of tumour and normal samples, respectively. The variable Δz is approximately normally distributed with a mean of zero and variance one. Thus, we are able to apply a Z test to calculate the associated *P*‐value $P_{\mathrm{ij}}^{z}$ under the null hypothesis that $z_{\mathrm{ij}}^{N}$ and $z_{\mathrm{ij}}^{T}$ are equal. The *P*‐values $P_{\mathrm{ij}}^{z}$ are controlled for multiple testing by BH correction. The weight of differential co‐expression between gene *i* and *j* is defined as:

(3) $$d_{\mathrm{ij}}=\left\{\begin{array}{cc} \left|\Delta z_{\mathrm{ij}}\right|, & \text{if}\;{\hat{P}}_{\mathrm{ij}}^{z}<t_{z}\;\text{and}\;\left(P_{\mathrm{ij}}^{N}<t_{N}\;\text{or}\;P_{\mathrm{ij}}^{T}<t_{T}\right)\\ 0, & \text{otherwise} \end{array}\right.$$

where ${\hat{P}}_{\mathrm{ij}}^{z}$ is the BH corrected value of $P_{\mathrm{ij}}^{z}$, and *t_z_* is the Z test *P*‐value threshold. Gene pairs with nonzero *d_ij_* were used to construct the DCN. A pair of genes is connected in the DCN if and only if they satisfy the following criteria: (a) the PCCs in normal and tumour samples are significantly different, and (b) the two genes are co‐expressed at a significant level in at least one group of samples. The DCN is a weighted graph with the edge weight reflecting the extent of differential co‐expression. The three thresholds *t_z_*, *t_N_* and *t_T_* control the reliability of the links in DCN. In general, the smaller the thresholds are, the more reliable the links are. We used *t_z_* = *t_N_* = *t_T_* = 10^−7^ in this study.

### 2.4 Scoring subnetworks

Both differential expression and differential co‐expression are integrated to score a subnetwork. Given a subnetwork *G* = (*V*, *E*), where *V* is the set of nodes, and *E* is the set of edges, the score of differential expression is defined as:

(4) $$DE_{G}=\frac{1}{\sqrt{\left|V\right|}}\sum_{i=1}^{\left|V\right|}\left|t_{i}\right|$$

where *t_i_* is the *t* statistic in a paired, two‐tailed *t* test comparing the expression values of gene *i* between tumour samples and normal samples, |*t_i_*| is the absolute value of *t_i_*, and |*V*| is the cardinality of the set *V*. The score of differential co‐expression is defined as:

(5) $$DC_{G}=\frac{1}{\sqrt{\left|E\right|}}\sum_{\left(i,j\right)\in E}d_{\mathrm{ij}}$$

where |*E*| is the cardinality of the set *E*. Then, Equations (4) and (5) are integrated to define the subnetwork score:

(6) $$D_{G}=\alpha DE_{G}+\left(1-\alpha \right)DC_{G}$$

where *α*∈[0,1] is a parameter to control the relative weight of differential expression *DE_G_* and differential co‐expression *DC_G_*.

### 2.5 Searching for lncRNA‐associated subnetworks

Given the subnetwork score function (6), a greedy search was performed to identify subnetworks within the DCN for which the scores are locally maximal.^28^ The search starts from a seed and iteratively adds neighbouring nodes. Each lncRNA is used as the seed in turn to initialize a subnetwork. At each iteration, the search considers addition of a node from the neighbours of nodes in the current subnetwork, and the corresponding edges connect this node and the current subnetwork. The addition which yields the maximum score is adopted. The search will stop if no node satisfies the following two conditions: (a) the number of edges in the shortest path between this node and the seed is less than or equal to *d*, and (b) the addition of this node increases the score of the subnetwork over an improvement rate *r*. The parameter *d* is a positive integer that controls the search space and *r* > 0 controls the increasing rate of the subnetwork score. For each lncRNA, the resulting subnetwork is usually composed of a few lncRNAs and a majority of PCGs, depending on its neighbouring genes.

### 2.6 Evaluation of subnetworks

Two tests were performed to evaluate the statistical significance of the subnetworks. The first one randomly permuted the sample labels 100 times and recalculated subnetwork scores of all real subnetworks. This generates a null distribution of random subnetwork scores for each real subnetwork. Then, the significance level *P*_1_ is obtained by indexing the score of each real subnetwork on the null distribution of the corresponding random subnetwork scores.

The second test constructed 100 random subnetworks for each real subnetwork. For a real subnetwork with *n* genes and *e* edges, the corresponding random subnetworks composed the seed lncRNA and other randomly selected *n*−1 genes. Edges among these *n* genes were sorted according to edge weight in decreasing order, and the top *e* edges were used to construct the random network. The subnetwork scores of these 100 random subnetworks constitute the null distribution of the real subnetwork score. Then, the significance level *P*_2_ is obtained by indexing the score of each real subnetwork on the corresponding null distribution.

### 2.7 Classification and clustering

The subnetwork expression profiles were inferred by the pathway activity inference method (DRWPClass) proposed by Liu et al.^29^ Three other methods (mean and median and PCA)^29^ were also performed for comparison. The logistic regression with lasso for feature selection was used to build the classifier, which was implemented with R package ‘glmnet’.^30^ Hierarchical clustering was performed with PCC as the distance measure and complete‐linkage as the clustering method.

### 2.8 Cell culture

Sources of oesophageal cancer cell lines have been described previously.^31^, ^32^ KYSE150, KYSE510 and TE3 cells were maintained in RPMI‐1640 medium containing 10% foetal bovine serum. KYSE450 cells were maintained in DMEM (HyClone) medium containing 10% newborn bovine serum. All were incubated with 5% CO_2_ and 80% humidity at 37°C.

### 2.9 Plasmid construction and transfection

Two *AL121899.1*‐expressing plasmids with a C‐terminal HA‐tag (AL121899.1‐HA) and GFP‐tag (pEGFP‐N1‐AL121899.1) were constructed by GENEWIZ. *ELMO2*‐expressing plasmids with N‐terminal GFP‐tag (C1‐ELMO2) were purchased from Sino Biological Inc. The corresponding empty vectors (pcDNA3.1‐C‐HA, pEGFP_N1 and pEGFP‐C1) were from our laboratory. KYSE150, KYSE510 or TE3 cells were seeded into plates and cultured for 16‐24 hours until 70% confluence. Plasmids were transfected into KYSE150, KYSE510 or TE3 cells using Lipofectamine 3000 (Invitrogen). Then, cells were cultured for 48 hours and used for further analysis.

### 2.10 RNA interference

Both *ELMO2* siRNA (siELMO2) and the scrambled siRNA (NC) were synthesized by GenePharma. The siRNA oligonucleotide sequences were as follows: siELMO2, 5′‐CCUUGAAAUCGACCAGAAATT‐3′ (sense), 5′‐UUUCUGGUCGAUUUCAAGGTT‐3′ (antisense); NC, 5′‐UUCUCCGAACGUGUCACGUTT‐3′ (sense), 5′‐ACGUGACACGUUCGGAGAATT‐3′ (antisense). KYSE450 or TE3 cells were seeded into plates and cultured for 16‐24 hours until 60%‐80% confluence. siRNA was transfected into KYSE450 or TE3 cells using Lipofectamine RNAiMAX reagent (Cat no. L13778‐150, Invitrogen) according to the manufacturer's transfection protocol and harvested at 48 hours post‐transfection.

### 2.11 Reverse transcription and quantitative real‐time PCR (qRT‐PCR)

Total RNA was extracted using TRIzol following the manufacturer's instructions. The concentration and purity were determined with a NanoDrop 2000 (Thermo). Total RNA (1 μg) was reverse transcribed into cDNA by a PrimeScript™ RT reagent kit with gDNA Eraser (Cat no. RR047B, TaKaRa) following the manufacturer's instructions. qRT‐PCR was performed using a SYBR Premix Ex Taq kit (TaKaRa) and using a 7500 Real‐Time PCR System (Applied Biosystems). Primer pairs used in the PCR analyses were as follows: *AL121899.1*, 5′‐CGTTTCTCCCGCGTCCTTCA‐3′ (forward), 5′‐AATGGTGCTCCTGCGTCACT‐3′ (reverse); *ELMO2*, 5′‐CCTGTTGCAGACATTAAGGCC‐3′ (forward), 5′‐GGTCTCATCAGGGTCATACAGG‐3′ (reverse); *β‐Actin*, 5′‐CAACTGGGACGACATGGAGAAA‐3′ (forward), 5′‐GATAGCAACGTACATGGCTGGG‐3′ (reverse). *β‐Actin* was used as the control and for normalization. All qRT‐PCR analyses for each gene were repeated at least three times.

### 2.12 Cell migration and proliferation assay

ESCC cells were transfected with plasmids or siRNA. For the transwell cell migration assay, cells were starved in serum‐free medium for 12 hours after being transfected for 36 hours, detached with EDTA solution and added to the top chamber at a density of 50 000 cells/well. The cells were incubated for 48 hours and the cells that migrated through the pores were fixed and stained with haematoxylin solution, and counted. For the wound healing assay, cells were seeded in a 6‐well plate, transfected with plasmids or siRNA at ~80% confluence and starved in serum‐free medium for 12 hours after being transfected for 36 hours. Then, the cells were incubated with 5% CO_2_ at 37°C after making straight scratches with a 200 μL yellow pipette tip. Images were taken using a 100× objective when there was difference between experimental group and control group. For MTS cell proliferation assay, cells were inoculated in each well of a 96‐well plate at 8 × 10^3^ cells/well. After 12 hours or 24 hours incubation, 20 μL MTL reagent (Promega) was added to each well, and cells were incubated 1‐2 hours at 37°C and subjected to colorimetric determination at 492 nm.

### 2.13 Western blotting assay

Western blotting was performed according to previously described methods.^33^ Briefly, total cell lysates were prepared in RIPA buffer, separated by SDS‐PAGE and transferred to PVDF membranes (Millipore). Membranes were incubated in blocking buffer and then incubated with the indicated antibody. Finally, immunoreactive bands were revealed using luminol reagent (Santa Cruz Biotechnology, DE). Photography and quantitative analyses were done using ChemiDoc Touch (Bio‐Rad). The following antibodies were used: mouse anti‐GFP (Santa Cruz Biotechnology) and mouse anti‐β‐actin (Sigma).

### 2.14 RNA sequencing

RNA‐Seq was applied to 12 samples (Table S1). *AL121899.1*‐expressing plasmids AL121899.1‐HA and pEGFP‐N1‐AL121899.1) were transfected into KYSE150 cells, respectively. The *ELMO2*‐expressing plasmid C1‐ELMO2 was transfected into KYSE510 cells. Correspondingly, three empty vector controls were transfected into their respective cell lines. Each was repeated once. Total RNA was extracted by TRIzol and deep sequenced on BGISEQ‐500 platform. RNA‐seq reads were mapped to the human reference genome (hg19) using bowtie2.^34^ Then, gene expression levels for each sample were calculated with RSEM.^35^

### 2.15 Statistical analysis

Statistical analyses were performed using R 3.4.2. The differentially expressed genes from the microarray data were defined as genes with a *t* test *P*‐value < .05 (BH correction) and a fold change > 2 or <0.5. The differential expression analysis of RNA‐Seq data was performed using DEGSeq.^36^ Gene enrichment analysis was performed using Metascape (http://metascape.org).^37^

## 3 RESULTS

### 3.1 Extensive loss of connectivity in the tumour co‐expression network

**Figure 1:**
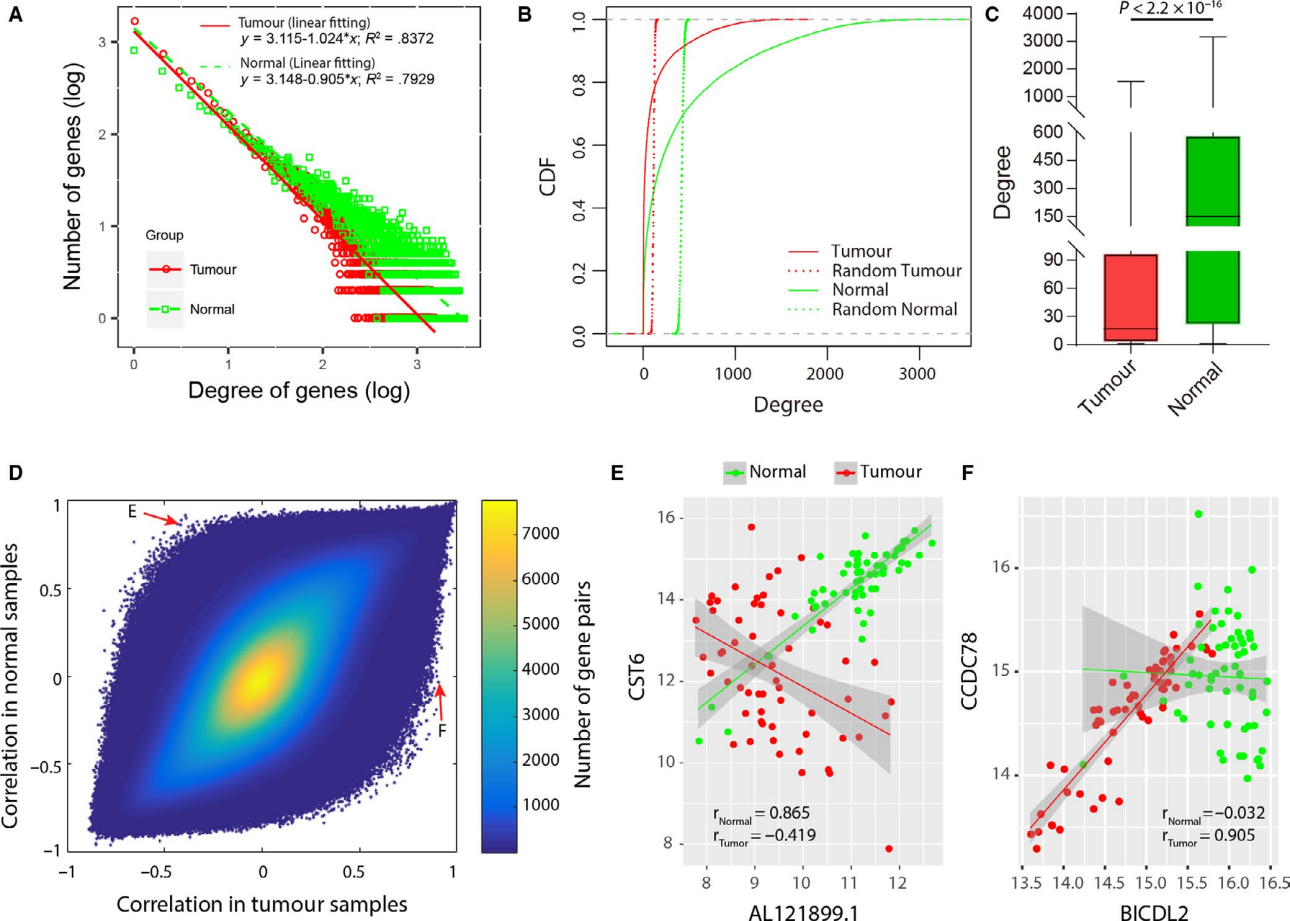
Comparison of the tumour co‐expression network (TCN) and normal co‐expression network (NCN). A, Scale‐free properties of degree distributions in the TCN and NCN. Numbers are shown on a log_10_ scale. B, Cumulative distribution functions (CDFs) of degrees of genes in the TCN and NCN. Compared with the random tumour network (red dotted line), the TCN had more genes with a large degree (red solid line). Similarly, the NCN had more genes with a large degree (green solid line) compared with the random normal network (green dotted line). C, Boxplots of degrees of genes in the TCN and NCN. Degrees of genes in the NCN were significantly larger than those in the TCN. D, Comparison of correlations in tumour samples and those in normal samples. E, Correlations of a gene pair (*AL121899.1* and *CST6*, located in the top left corner in (D)) in normal and tumour samples. F, Correlations of a gene pair (*BICDL2* and *CCDC78*, located in the bottom right corner in (D)) in normal and tumour samples

The data set ESCC‐train was used to construct co‐expression networks. Genes with invariable expression across 60 paired ESCC and adjacent normal tissues (coefficient of variation < 0.05) were filtered out, resulting in 13 073 PCGs and 5379 lncRNAs. Based on the 60 normal tissues, the PCC and the corresponding Student's *P*‐value of each gene pair were calculated. Then, an NCN was constructed using the gene pairs with BH‐adjusted *P*‐values < 10^−7^. A TCN was constructed based on the 60 ESCC tissues in the same way. The *P*‐value threshold of 10^−7^ corresponds to a PCC of 0.683 (or −0.683) and 0.704 (or −0.704) in NCN and TCN, respectively. Both NCN and TCN were scale‐free networks with the degree distribution following a power law (Figure 1A). Compared with degree‐preserving random networks constructed using the Erdos‐Renyi model,^38^ the two networks were characterized by nodes with highly variable degrees, from a few to thousands (Figure 1B). The cumulative distribution functions (CDF) of degrees of NCN and TCN deviated from those of the corresponding random normal networks and tumour networks, respectively (Figure 1B, *P* < 2.2 × 10^−16^, Kolmogorov‐Smirnov test). Both NCN and TCN had large clustering coefficients (*c*) of 0.4709 and 0.4600, and small average shortest path lengths (*L*) of 3.0652 and 3.9116, respectively (Table 1). In the corresponding random networks, the average clustering coefficients were 0.0272 and 0.0110 (Figure S1A), and average shortest path lengths were 1.9728 and 2.2732 (Figure S1B), respectively, indicating that the two co‐expression networks had all the properties of a scale‐free, small‐world network (*c*»*c_random_*, *L*≈*L_random_*).^39^ The high clustering coefficients and their distributions of approximate scaling law *c*(*k*)~*k* ^−1^ (Figure S1C and D) suggested hierarchical modularity in both NCN and TCN that is typical for cellular networks.^40^, ^41^

**Table 1:** Characteristics of the normal co‐expression network (NCN), tumour co‐expression network (TCN) and differential co‐expression network (DCN)

	NCN	TCN	NCN∩TCN	NCN\TCN^a^	DCN
No. of genes	15 424	10 345	7726	7698 (49.91%)	2074
PCGs	11 482	7640	5756	5726 (49.87%)	1746
lncRNAs	3942	2705	1970	1972 (50.03%)	328
No. of edges	3 236 238	587 517	345 016	2 891 267 (89.34%)	3917
PCG‐PCG	2 030 506 (62.74%)	254 673 (43.35%)	147 452 (42.74%)	1 883 054 (92.74%)	3277 (83.66%)
PCG‐lncRNA	1 001 184 (30.94%)	240 326 (40.91%)	139 705 (40.49%)	861 479 (86.05%)	618 (15.78%)
lncRNA‐lncRNA	204 548 (6.32%)	92 518 (15.75%)	57 859 (16.77%)	146 653 (71.70%)	22 (0.56%)
Mean degree	419.64	113.58			3.78
Median degree	152	17			1
Maximum degree	3160	1547			327
Mean clustering coefficient	0.4709 [0.027]^b^	0.4600 [0.011]			0.0275 [0.002]
Diameter of the network	12 [3]	20 [3]			18 [12]
Mean shortest path length	3.065 [1.973]	3.912 [2.273]			5.636 [5.841]

^a^ Genes/edges in NCN but not TCN. The proportions in the column NCN\TCN were the percentages of the number of genes/edges in NCN\TCN to those in NCN.

^b^ Properties of degree‐preserving random networks.

Consistent with other cancer types,^12^, ^20^ for ESCC, the TCN also displayed a reduced connectivity compared with the NCN (Figure 1A‐C). In the NCN, there were 11 482 PCGs and 3942 lncRNAs that were linked by 3 236 238 edges. There were only 7640 PCGs and 2705 lncRNAs that were linked by 587 517 edges in the TCN. The degrees of genes in the NCN were significantly larger than those in the TCN (median degree 152 vs 17, *P* < 2.2 × 10^−16^, Wilcoxon rank sum test, Figure 1C). About 89.34% of the edges in the NCN were not conserved in the TCN, especially those connecting PCGs (92.74%, Table 1), suggesting a seriously disrupted regulatory system in tumour samples.

### 3.2 Construction of the DCN in ESCC

**Figure 2:**
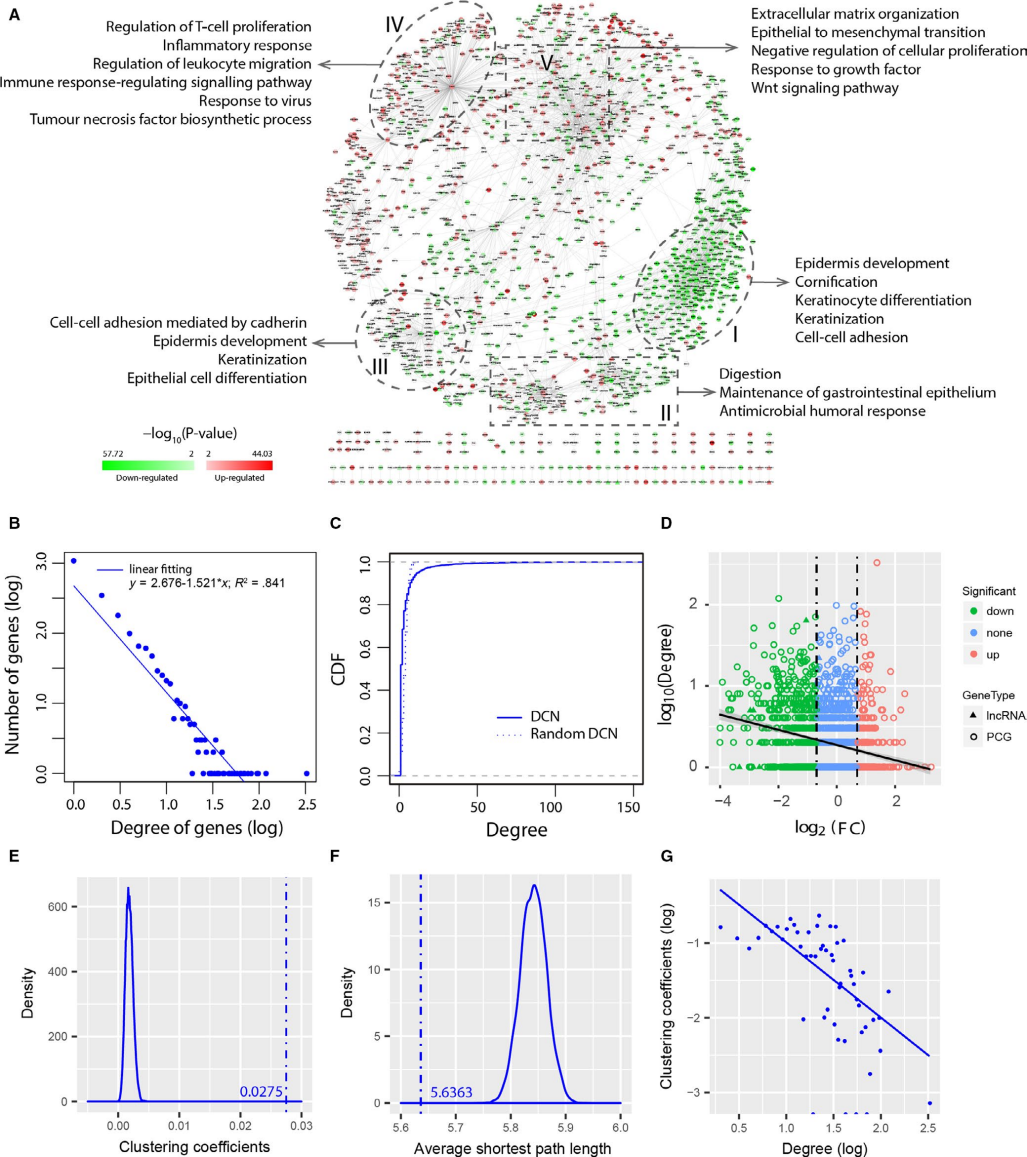
Differential co‐expression in ESCC. A, Visualization of the differential co‐expression network (DCN). Circle nodes represent PCGs, and triangle nodes represent lncRNAs. Node colour represents differential expression level. Green represents that the gene is down‐regulated. Red represents that the gene is up‐regulated. Regions I‐V contain PCGs that are enriched in various GO annotations. B, Scale‐free property of degree distribution in the DCN. The blue line depicts the least‐squares fit of the data to a linear line. C, Comparison of the CDF of degrees in the DCN and in a random network with the same number of nodes and edges as the DCN. D, Relation of differential expression and differential co‐expression. Shown is the fold change (*x*‐axis) versus degree in the DCN (*y*‐axis). Degrees of genes in the DCN had weak correlation with their fold changes. E, Distribution of average clustering coefficients for random networks. The average clustering coefficient (*c*) of the DCN is much larger than those of random networks (*c*»*c_random_*). F, Distribution of average shortest path lengths for random networks. The average shortest path length (*L*) of the DCN is comparable to those of random networks (*L* ≈ *L_random_*). G, Distribution of clustering coefficients in the DCN, which follows the scaling law *c*(*k*)~*k* ^−1^

Inspired by the difference between the NCN and TCN, we compared the PCCs of all gene pairs in normal samples and tumour samples (Figure 1D). Most gene pairs had consistent PCCs between the two conditions. Nevertheless, the correlations of some gene pairs differed markedly between normal and tumour samples (top left and bottom right corner in Figure 1D). For example, lncRNA *AL121899.1* and PCG *CST6* were positively correlated in normal samples (*r*_Normal_ = .856), but negatively correlated with tumour samples (*r*_Tumour_ = −.419, Figure 1E). While *BICD2* and *CCDC78* had no correlation in normal samples, they were positively correlated in tumour samples (*r*_Tumour_ = .905, Figure 1F). To measure the significance of the difference between PCCs in normal and tumour samples, a Fisher transformation followed by a *Z* test was applied to the PCCs. A strict *P*‐value threshold of 10^−7^ was used to determine whether the PCCs in normal and tumour samples were significantly different. Then, a gene pair was considered as differentially co‐expressed if the *P*‐value in the *Z* test < 10^−7^ and simultaneously, the two genes were connected in the NCN or TCN. Finally, a DCN was constructed based on differentially co‐expressed gene pairs (Figure 2A).

In the DCN, there were 1746 PCGs and 328 lncRNAs that were linked by 3917 edges. As NCN and TCN, DCN was a scale‐free network with highly variable degrees (Figure 2B). The degree distribution of the DCN was different from that of a degree‐preserving random network (Figure 2C, *P* < 2.2 × 10^−16^, Kolmogorov‐Smirnov test). The degrees of PCGs were significantly larger than those of lncRNAs (mean degree 4.11 vs 2.02; Wilcoxon rank sum test, *P* = 7.2 × 10^−9^). Of the 1746 PCGs in the DCN, 1696 (97.14%) were annotated with at least one GO term, most commonly (1542, 88.32%) with a GO BP term. However, only 35.10% (728, 648 PCGs and 80 lncRNAs) of the genes were differentially expressed. This is consistent with previous studies, which found that differential expression sheds little light on differential co‐expression.^20^, ^21^ In 29.45% of the links, neither of the two genes was differentially expressed. Genes with high connections were evenly distributed across down‐regulated, non‐significant and up‐regulated genes (Spearman correlation coefficient = −.21, Figure 2D), indicating that differential co‐expression had weak correlation with differential expression.

The average clustering coefficient of the DCN was much larger than that of the random networks (*c_DCN_* = 0.0275» *c_random_* ≈ 0.0018, Figure 2E). The shortest path length was comparable to that of the random networks (*L_DCN_* = 5.6363 ≈ *L_random_* ≈ 5.8414, Figure 2F), indicating that the DCN is also a small‐world network.^39^ Furthermore, the clustering coefficients asymptotically followed the scaling law *c*(*k*)~*k* ^−1^ (Figure 2G), suggesting that the DCN is characterized by a potential modular organization, which is a general feature of biological networks.^40^, ^41^ This inspired us to question whether the topological modularity of the DCN could reflect the true functional organization or molecular mechanisms underlying the development of ESCC. On a global perspective, genes in the DCN formed several clusters that were enriched in multiple GO annotations (Figure 2A). Down‐regulated genes tended to cluster together (eg Clusters I and III), as well as up‐regulated genes (eg Cluster IV). The vast majority of genes in Cluster I were down‐regulated. The PCGs in Cluster I were enriched in GO BP terms concerning epidermis development (*P* = 5.47 × 10^−19^), cornification (*P* = 1.24 × 10^−17^) and keratinocyte differentiation (*P* = 2.69 × 10^−15^). In contrast, the majority of genes in Cluster IV were up‐regulated. The PCGs in Cluster IV were enriched in many GO BP terms that are frequently altered in cancer, such as regulation of T‐cell proliferation (*P* = 8.91 × 10^−8^) and inflammatory response (*P* = 2.02 × 10^−6^). In addition, the PCGs in Cluster II were enriched in GO BP terms associated with the digestive system process. A bird's‐eye view of the DCN suggested that the DCN could help reveal functional modules that play important roles during the development of ESCC.

### 3.3 Identification of lncRNA‐associated differential subnetworks

To identify lncRNA‐associated differential subnetworks, a greedy search algorithm was applied to search differential subnetworks in the DCN using each of the 328 lncRNAs as the seed. A total of 328 lncRNA‐associated subnetworks were identified. With the parameters *d* = 2, *r* = .1 and *α* = .7, the number of nodes in the identified subnetworks ranged from two to 14 (mean number = 7.45), with the majority (63.72%) having between six and 10 nodes (Figure S2A). The number of edges ranged from one to 19 (mean number = 6.98) and the majority (58.23%) between five and nine (Figure S2B). The subnetwork scores had a mean of 16.44 and did not increase with the number of genes when the number of genes exceeded 5 (Figure S2C) and also the number of edges when the number of edges exceeded 4 (Figure S2D), indicating that the subnetwork scores were independent of the sizes of subnetworks.

To identify differential subnetworks that are statistically significant, two tests of statistical significance were performed to assess the 328 subnetworks. About 98.48% (323/328) of the subnetworks passed the first test (*P*_1_ < .05), while 53.96% (177/328) of the subnetworks passed the second test (*P*_2_ < .05). In total, 177 subnetworks that passed both tests were considered statistically significant in the ESCC‐train set. To identify robust subnetworks that are consistently significant across data sets, we further assessed the significance of the 328 subnetworks in both the ESCC‐test set and the ESCC‐valid set. The subnetwork scores were recalculated based on gene expression values in the new data sets and subjected to the two tests. Of the 328 subnetworks, 147 and 117 subnetworks were significant in the ESCC‐test set and the ESCC‐valid set, respectively. Finally, 107 subnetworks that were significant in all three data sets were considered to be differential subnetworks (Table S2‐S3). Figure S3 illustrates an example of differential subnetwork (DS_*AL121899.1*), which was identified by using lncRNA *AL121899.1* as the seed. DS_*AL121899.1* was a down‐regulated subnetwork located in Region I in the DCN (Figure 2A). All nine genes in DS_*AL121899.1* were down‐regulated, and 11 gene pairs were positively correlated in the normal samples but lost the correlations in the tumour samples. As expected, the differential expression (Figure S3B) and differential co‐expression patterns (Figure S3C) were consistent across the three data sets, indicating the robustness of the differential subnetworks.

### 3.4 Differential subnetworks are discriminative between ESCC and normal tissues

**Figure 3:**
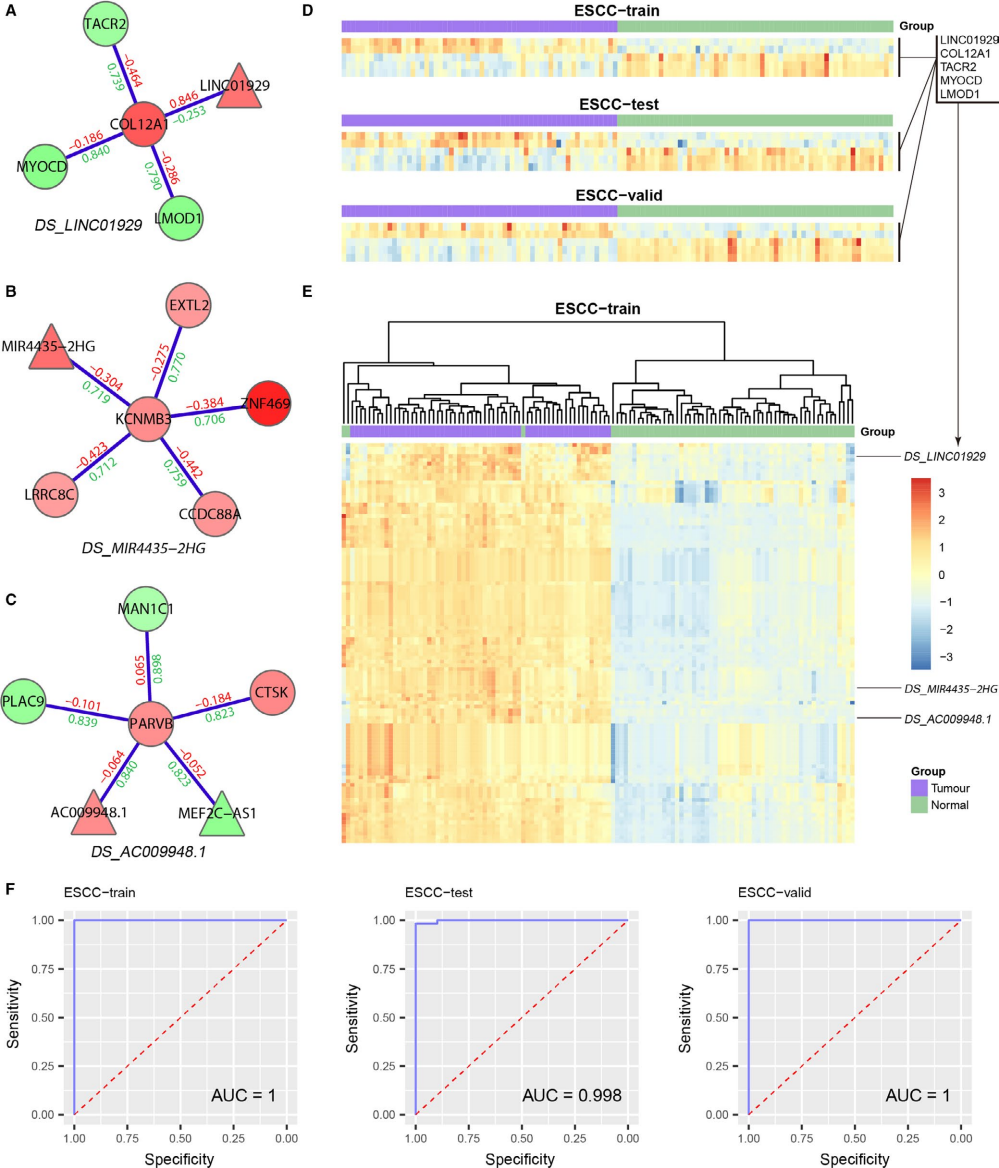
Discrimination of differential subnetworks. Three discriminative subnetworks are shown in (A‐C). D, Consistent expression patterns of the five genes in DS_*LINC01929*. E, Heatmap of subnetwork expression profiles in the ESCC‐train. F, Classification performance of the discriminative subnetworks

Given that the differential subnetworks showed consistently distinct expression patterns between ESCC and normal samples, we next investigated whether differential subnetworks were discriminative to distinguish ESCC from normal tissues. To this end, a network‐based classifier called DRWPClass was constructed based on the ESCC‐train and then evaluated on the ESCC‐test and ESCC‐valid. For each differential subnetwork, DRWPClass integrated expression values of the gene members into a subnetwork expression value. For example, in the differential subnetwork DS_*LINC01929* (Figure 3A), two up‐regulated genes (*LINC01929* and *COL12A1*) and three down‐regulated genes (*TACR2*, *MYOCD* and *LMOD1*) had consistent expression patterns across the three data sets (Figure 3D). Expression profiles of the five genes in the ESCC‐train were integrated into a subnetwork expression profile, which corresponded to one row in the heatmap of the subnetwork expression profiles (Figure 3E). Two other discriminative subnetworks (DS_*MIR4435‐2HG*, Figure 3B; DS_*AC009948.1*, Figure 3C) were also marked in Figure 3E. Clustering analysis showed that 107 subnetwork expression profiles could distinguish ESCC from normal samples across all three data sets (Figure 3E, Figure S4 and S5). Feature selection with lasso identified 11 discriminative subnetworks (Table S4), which almost perfectly classified ESCC and normal samples in the three data sets (AUC = 1, 0.998 and 1, respectively, Figure 3F). Three other pathway activity inference methods (mean, median and PCA) identified different subnetwork markers (Table S4). According to the principle of the pathway activity inference methods, the differential subnetworks identified by the mean and median method tend to contain genes that are simultaneously up‐regulated or down‐regulated (eg DS_*MIR4435‐2HG*, Figure 3B), while differential subnetworks identified by the PCA method tend to have the largest overall variations in gene expression. The differential subnetworks identified by DRWPClass have the strongest discriminative ability (Table S4), including not only the subnetworks with consistent gene expression changes, but also the subnetworks containing both up‐regulated and down‐regulated genes (eg DS_*AC009948.1*, Figure 3C), which are common in dysfunctional regulatory networks. However, all the four methods yielded favourable classification performances (Table S5), suggesting that all these subnetworks are discriminative to serve as potential subnetwork biomarkers.

### 3.5 Core differential co‐expression modules

**Figure 4:**
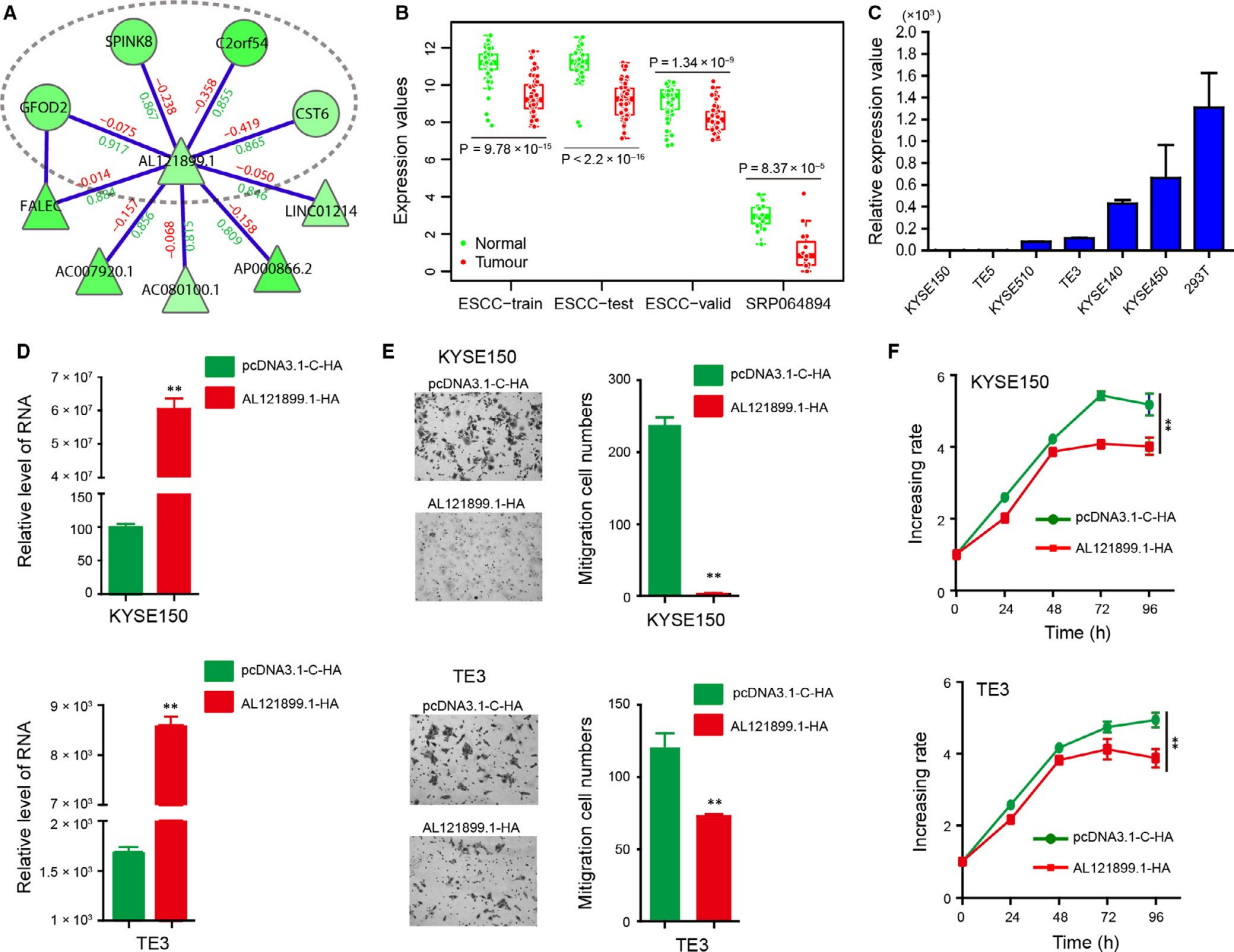
AL121899.1‐associated core module. A, Visualization of the AL121899.1‐associated core module. Region in the elliptic curve is the frequently identified core module. B, Comparison of AL121899.1 expression between normal and tumour samples in multiple data sets. C, qRT‐PCR assay detects AL121899.1 expression in multiple ESCC cell lines, using 293T cells as the control. D, qRT‐PCR assay detects AL121899.1 expression in KYSE150 and TE3 after HA‐AL12899.1 transfection. E, Transwell migration assays analyse the effects of AL121899.1 overexpression on cell migration in KYSE150 and TE3 cells. F, MTS experiments analyse the effects of AL121899.1 overexpression on cell proliferation in KYSE150 and TE3 cells. **P < .01

**Figure 5:**
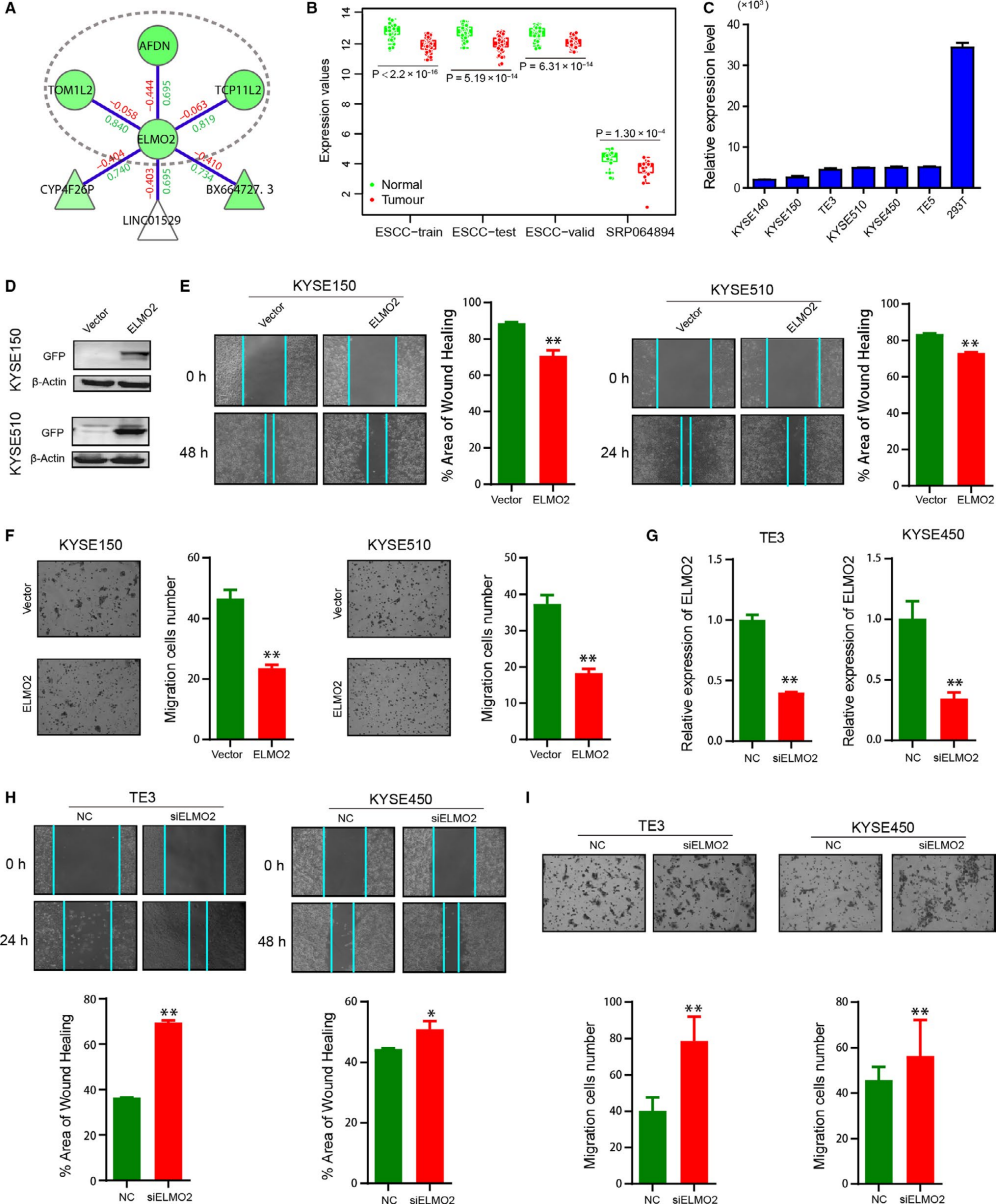
ELMO2‐associated core module. A, Visualization of ELMO2‐associated core module. Region in the elliptic curve is the frequently identified core module. B, Comparison of ELMO2 expression between normal and tumour samples in multiple data sets. C, qRT‐PCR assay detects ELMO2 expression in multiple ESCC cell lines, using 293T cells as the control. D, Analysis of ELMO2 expression, after GFP‐ELMO2 transfection, by immunoblotting. E, Wound healing assays to analyse the effects of ELMO2 overexpression on cell migration in KYSE150 and KYSE510 cells. F, Transwell migration assays to analyse the effects of ELMO2 overexpression on cell migration in KYSE150 and KYSE510 cells. G, qRT‐PCR detects ELMO2 expression in TE3 and KYSE450 cells after siELMO2 transfection. H, Wound healing assays to analyse the effects of ELMO2 knockdown on cell migration in TE3 and KYSE450 cells. I, Transwell migration assays to analyse the effects of ELMO2 knockdown on cell migration in TE3 and KYSE450 cells. *P < .05, **P < .01, ***P < .001

Among 107 differential subnetworks, some subnetworks overlapped. Genes located in the overlapping regions were frequently captured by differential subnetworks. We speculated that these genes may play important roles in ESCC and are worth paying more attention. Thus, we applied a greedy search to identify overlapping gene modules (referred to as core differential co‐expression modules) across all the differential subnetworks. Four core modules with ≥4 genes and captured by ≥3 differential subnetworks were identified, including and *AL121899.1*‐associated core module (Figure 4A), an *ELMO2*‐associated core module (Figure 5A), and a *BICDL2‐* and *KRT78*‐associated core module (Figure S6). Two of them were further investigated in the following section.

#### 3.5.1 *AL121899.1*‐associated core module

The *AL121899.1*‐associated core module was identified in six differential subnetworks (DS_*AL121899.1*, DS_*FALEC*, DS_*AC007920.1*, DS_*AC080100.1*, DS_*AP000866.2* and DS_*LINC01214*). All five genes in the module (*AL121899.1*, *GFOD2*, *SPINK8*, *C2orf54* and *CST6*) were down‐regulated and lost positive correlations with their neighbours in tumour samples. *C2orf54* was consistently down‐regulated in multiple independent data sets and has been reported as a potential ESCC biomarker.^42^ *SPINK8* was suggested to be a tumour suppressor gene, since transfecting *SPINK8* into ESCC cell line EC9706 inhibits cell proliferation and migration, and promotes cell apoptosis.^43^ *CST6* suppresses tumour cell growth through cytoplasmic retention of NF‐κB.^44^ It belongs to the *CST* superfamily whose members have been shown to be associated with the metastasis and invasiveness of several tumours.^45^, ^46^ Loss of *CST6* expression depends on its promoter hyper‐methylation in metastatic breast cancer cell lines.^47^ Interestingly, *CST6* is also down‐regulated and promoter hypermethylated in ESCC,^48^ suggesting a similar mechanism in ESCC. In fact, of the 56 down‐regulated genes with promoter hyper‐methylation in ESCC reported by Otsubo et al,^48^ 44.6% (25/56) were captured by the DCN, including *C2orf54* in the core module. Most of them (22/25) are located in Region I in the DCN, suggesting that hyper‐methylation may have a role in differential co‐expression between down‐regulated genes.

As the hub of the core module, *AL121899.1* is a lncRNA whose function has not been clarified. We speculated that *AL121899.1* may play important functions in ESCC. It had a maximum degree in the DCN and was consistently down‐regulated in multiple data sets, including the ESCC‐train (*P* = 9.78 × 10^−15^), ESCC‐test (*P* < 2.2 × 10^−16^), ESCC‐valid (*P* = 1.34 × 10^−9^) and SRP064894^10^ (*P* = 8.37 × 10^−5^, Figure 4B). qRT‐PCR assays further confirmed the low expression of *AL121899.1* in ESCC cells, such as KYSE150, TE5, KYSE510 and TE3 (Figure 4C). To investigate the potential functions of *AL121899.1*, we over‐expressed *AL121899.1* in both KYSE150 and TE3 cells by HA‐*AL121899.1* transfection (Figure 4D). Transwell migration assays showed that *AL121899.1* overexpression significantly reduced ESCC cell migration in both KYSE150 and TE3 cells (*P* < .01, Figure 4E). Moreover, MTS experiments showed that *AL121899.1* overexpression also inhibited ESCC cell proliferation (Figure 4F).

Furthermore, we transfected *AL121899.1* into KYSE150 cells and investigated gene expression changes from RNA‐Seq. The high correlation coefficients between sample repeats confirmed the quality of RNA‐Seq (Figure S7A). Compared with the empty vector controls, 119 genes were up‐regulated and 53 down‐regulated in the samples overexpressing *AL121899.1* (Table S6‐S7, Figure S7B). Overexpressing *AL121899.1* did not affect the expression of genes in the core module, but instead regulated a set of genes enriched on hallmark epithelial‐mesenchymal transition and other 19 immunologic or oncogenic signatures in MSigDB v6.2 (Table S8),^49^ and also many genes that have been reported to be associated with prognosis, metastasis, chemoresistance or radioresistance of ESCC, such as SPARC,^50^, ^51^ FSTL1,^52^ MUC4,^53^ DHCR7,^54^ LOX^53^ and CXCL1.^55^ In addition, the up‐regulated genes were enriched on biological processes associated with regulation of insulin‐like growth factor transport and uptake (*P* = 1.43 × 10^−6^), post‐translational protein phosphorylation (*P* = 8.70 × 10^−6^), steroid metabolic process (*P* = 1.32 × 10^−5^) and digestion (*P* = 3.09 × 10^−3^) (Figure S7C, Table S9).

#### 3.5.2 *ELMO2*‐associated core module

As for *AL121899.1*, *ELMO2* is the hub of the *ELMO2*‐associated core module. It was consistently down‐regulated in the ESCC‐train (*P* < 2.2 × 10^−16^), ESCC‐test (*P* = 5.19 × 10^−14^), ESCC‐valid (*P* = 6.31 × 10^−14^) and SRP064894 (*P* = 1.30 × 10^−4^, Figure 5B). qRT‐PCR assays showed that *ELMO2* had low expression in ESCC cells, using 293T cells as the control (Figure 5C), suggesting an important role in ESCC. Thus, we sought to investigate the potential functions of *ELMO2*. Firstly, we transfected GFP‐*ELMO2* into KYSE150 and KYSE510 cells, and confirmed *ELMO2* expression by immunoblotting (Figure 5D). Both wound healing assays and transwell migration assays showed that *ELMO2* overexpression reduced cell migration (Figure 5E,F). Secondly, we knocked down *ELMO2* in TE3 and KYSE450 cells by siELMO2 transfection, and confirmed *ELMO2* expression by qRT‐PCR (Figure 5G). In contrast to *ELMO2* overexpression, *ELMO2* knockdown promoted cell migration (Figure 5H,I). These results indicate that ELMO2 inhibits cell migration in ESCC.

Furthermore, we transfected *ELMO2* into KYSE510 cells and investigated gene expression changes from RNA‐Seq. The high correlation coefficients between sample repeats confirmed the quality of RNA‐Seq (Figure S8A). Compared with the empty vector controls, 108 genes were up‐regulated and 147 down‐regulated in the samples overexpressing *ELMO2* (Table S10 and S11, Figure S8B). The up‐regulated genes were enriched on biological processes associated with cell‐cell adhesion, interferon‐gamma production, acute inflammatory response and cytokine‐cytokine receptor interaction (Figure S8C). The down‐regulated genes were enriched on biological processes associated with axoneme assembly and membrane repolarization (Figure S8D).

In all, the functional experiments showed that *AL121899.1* and *ELMO2* are two important tumour suppressors in ESCC. This indicates that the differential subnetworks could suggest reliable targets for further study.

## 4 DISCUSSION

In this study, we investigated the topological characteristics of the DCN in ESCC. As with other cellular networks, the DCN is a scale‐free, small‐world network. These topological characteristics were not dependent on the method used to construct the DCN, as DCNs constructed by a different method DCe^56^ with different cut‐offs also had the properties of *c*»*c_random_* and *L*≈*L_random_* (Figure S9). The high clustering coefficient implies modular organization of the network,^40^ which implies the genes in the lncRNA‐associated differential subnetworks may work in a modular manner to contribute to the development of ESCC.

Differential co‐expression analysis has been used to improve functional enrichment analysis,^15^ unveil differential regulation^16^ and detect differentially co‐expressed clusters globally, such as WGCNA,^17^ DiffCoEx^18^ and DICER.^19^ Different from these studies, our subnetwork searching algorithm focuses on identifying differential co‐expressed subnetworks associated with a specific node, for example a lncRNA. With the parameters *d* = 2, *r* = .1 and *α* = .7, the identified subnetworks had moderate sizes that were convenient for further analysis and functional validation (Figure S2A,B). The subnetwork size can be controlled by adjusting the parameters *r* and *d* in the algorithm. The parameter *r* controls the increasing rate of the subnetwork score. A large *r* will prevent the addition of genes that could not yield enough improvement on the subnetwork score. Thus, the larger the *r*, the smaller the subnetwork size. This was demonstrated by rerunning the subnetwork searching algorithm with different *r* values (*r* = .05, .07, .1, .2 and .3). With the increase of *r*, the number of nodes, the number of edges and the subnetwork scores were decreased (Figure S10A‐C). At the same time, the identified subnetworks tended to be more significant as the addition of a new gene at each iteration becomes stricter. Similarly, the parameter *d* controls search space. The subnetwork size increases with the increase of *d* (Figure S10D‐F). Another characteristic of our method is that it integrates both differential expression and differential co‐expression for subnetwork identification. The relative weight of differential expression and differential co‐expression is controlled by the parameter *α*. When *α* approaches 1, our method will be reduced to a differential expression‐based method except for the underlying differential co‐expression network (Formula (6)). The parameter *α* does not affect the subnetwork size much (Figure S10G‐I), but increases differential expression scores and reduces differential co‐expression scores when it gives more weight on differential expression (Figure S10J,K). These results suggest that, compared to differential expression‐based methods, our method focuses on identifying biologically meaningful subnetworks at the cost of some discriminability. However, it is just an indirect comparison with differential expression‐based methods based on our differential co‐expression network. To objectively evaluate our method, rigorous comparisons with similar methods that also incorporate differential co‐expression scores are needed in the future.

Differential co‐expression analysis has been reported to be able to suggest new biomarker candidates and provide novel hypotheses for specific functional experiments.^12^ LncRNAs and PCGs in a same differential subnetwork may work together to perform specific functions. Except for *AL121899.1 and ELMO2*, whose abilities to inhibit tumour growth were confirmed by functional experiments, many other genes in the core modules have been reported as tumour suppressor genes, such as *SPINK8*, *CST6* and *C2orf54*,^42^, ^43^, ^44^ highlighting the ability of the core modules to indicate candidate cancer genes.

Differential co‐expression is complementary to differential expression for depicting dysregulated systems in cancer.^12^, ^20^ Differential co‐expression analysis has successfully identified driver genes that could not be found by differential expression analysis.^20^, ^57^ In the DCN, genes that were not differentially expressed also had the possibility to play a role in ESCC due to their differential connections. For example, *DLC1* is a known tumour suppressor gene that may be involved in the carcinogenesis of ESCC.^58^ It was captured in the DCN, but missed in differential expression analysis as it exhibits no differential expression (*P* = .078). In the *KRT78‐* and *BICDL2*‐associated core module, the two hubs were connected by 15 lncRNAs (Figure S6). Although many of them were not differentially expressed, they were differentially co‐expressed with both *KRT78* and *BICDL2*. Among these genes, lncRNA *C20orf204* (named *LINC00176* in GRCh37 coordinates, *P* = .168) has been confirmed to negatively regulate cell proliferation in Huh7.5_OC_ cells.^59^ Tran et al found that *C20orf204* regulates expression of more than 200 genes by a sponge function for tumour suppressor miRNAs in hepatocellular carcinoma.^60^ Its function in ESCC is also worth further investigation. Other top‐ranked genes with a high degree include *TMTC1*, *OLFM1*, *TRIM31* and *FGF13*.

In summary, we identified a source of lncRNA‐associated differential subnetworks on a large scale by integrating differential expression and differential co‐expression. The functional experiments on *AL121899.1* and *ELMO2* confirmed the effectiveness of the subnetwork identification method. The differential subnetworks will be helpful for revealing the dysfunctional regulatory networks of ESCC and generating hypotheses for the discovery of novel gene or subnetwork biomarkers. However, identification of differential subnetworks is the first step towards understanding the dysfunctional regulatory systems in the development of ESCC. Further analyses are needed to illustrate the detailed regulatory mechanisms underlying the differential subnetworks in the future. The proposed subnetwork identification method has been implemented as an R package ‘DCN’ (https://github.com/weiliu123/DCN-package), which can be easily used to investigate differential subnetworks of other molecules in other cancers.

## CONFLICT OF INTEREST

The authors confirm that there are no conflicts of interest.

## AUTHOR CONTRIBUTIONS

EML and LYX conceived the concept for this study; WL designed the algorithm and performed the bioinformatic analyses; CYG and LDL carried out the biological experiments; WW and CQL performed network analysis; WL and CYG wrote the manuscript; EML and LYX revised the manuscript.

## Supporting information

Supplementary Material

## Data Availability

All other data supporting the presented findings are available from the corresponding author upon request.
