# Supplementary material for: Identification of lncRNA‐associated differential subnetworks in oesophageal squamous cell carcinoma by differential co‐expression analysis
Source: J Cell Mol Med. 2020 Mar 12;24(8):4804–18. doi: 10.1111/jcmm.15159 (PMC7176870; doi:10.1111/jcmm.15159)
Supplement: Supplementary file 1 — Supplementary Material [file JCMM-24-4804-s001.docx]

**Figure S1.** Topological characteristics of the normal co-expression network (NCN) and tumor co-expression network (TCN). (**A**) Distributions of average clustering coefficients for random normal (green) and tumor (red) networks. The average clustering coefficients (*c*) of the NCN and TCN are much larger than those of the corresponding random networks (*c* >> *c_random_*). (**B**) Distributions of average shortest path lengths for random normal (green) and tumor (red) networks. The average shortest path lengths (*L*) of NCN and TCN are comparable to those of the corresponding random networks (*L* ≈ *L_random_*). (**C**) Distribution of clustering coefficients in the NCN. (**D**) Distribution of clustering coefficients in the TCN.

**Figure S2.** Characteristics of the 328 subnetworks. (**A**) Distribution of the number of nodes in the 328 subnetworks. (**B**) Distribution of the number of edges in the 328 subnetworks. (**C**) Boxplot of subnetwork scores grouped by the number of genes. (**D**) Boxplot of subnetwork scores grouped by the number of edges.

**Figure S3.** Expression patterns of genes in DS_*AL121899.1* across three datasets. (**A**) Visualization of DS_*AL121899.1*. Circle nodes represent PCGs, and triangle nodes represent lncRNAs. Node color represents differential expression level. Green indicates the gene is down-regulated. (**B**) Heatmaps of the expression values of nine genes in DS_*AL121899.1*. (**C**) Expression patterns of 11 edges were consistent across three datasets.

**Figure S4. Heatmap of subnetwork expression profiles in the ESCC-test.**

**Figure S5. Heatmap of subnetwork expression profiles in the ESCC-valid.**

**Figure S6. *KRT78-* and *BICDL2*-associated core module.** Regions in the elliptic curve are frequently identified core modules.

**Figure S7. Functional expression analysis of *AL121899.1*.** (**A**) Correlation analysis between eight samples. (**B**) Hierarchical clustering of differential genes. (**C**) Enrichment analysis of up-regulated differential genes.

**Figure S8. Functional expression analysis of *ELMO2*.** (**A**) Correlation analysis between four samples. (**B**) Hierarchical clustering of differential genes. (**C**) Enrichment analysis of up-regulated differential genes. (**D**) Enrichment analysis of down-regulated differential genes.

**Figure S9. Topological properties of differential co-expression networks (DCNs) constructed by DCe.** (**A-B**) DCN was constructed by DCe with cutoff=p=0.01. (**A**) Distribution of average clustering coefficients for random networks. The average clustering coefficient (*c*) of the DCN is much larger than those of random networks (*c* = 0.0122 >> *c_random_* = 0.0025). (**B**) Distribution of average shortest path lengths for random networks. The average shortest path length (*L*) of the DCN is comparable to those of random networks (*L*=3.22 ≈ *L_random_*=3.17). (**C-D**) DCN was constructed by DCe with cutoff=p=0.001. (**C**) The average clustering coefficient of the DCN is much larger than those of random networks (*c* = 0.0033 >> *c_random_* = 0.0004). (**D**) Distribution of average shortest path lengths for random networks. The average shortest path length of the DCN is comparable to those of random networks (*L*=7.57 ≈ *L_random_*=9.10).

**Figure S10. Analysis of the parameters in the subnetwork searching algorithm.** (**A**-**C**) With the increase of *r*, the number of genes (**A**), the number of edges (**B**), and network scores (**C**) decreased. (**D**-**F**) With the increase of *d*, the number of genes (**D**), the number of edges (**E**), and network scores (**F**) increased. (**G**-**K**) The influence of alpha on the number of genes (**G**), the number of edges (**H**), subnetwork scores (**I**), DE scores (**J**) and DC scores (**K**).

**Table S1**. Twelve samples sequenced by the BGISEQ-500 platform.

**Table S2**. The 107 significant differential subnetworks.

**Table S3**. The 107 significant differential subnetworks.

**Table S4**. Discriminative differential subnetworks identified by lasso.

**Table S5**. AUCs of pathway activity inference methods.

**Table S6**. Up-regulated genes in samples with *AL121899.1* overexpression.

**Table S7**. Down-regulated genes in samples with *AL121899.1* overexpression.

**Table S8**. Terms in MSigDB enriched by genes mediated by *AL121899.1* overexpression.

**Table S9**. Enrichment analysis of upregulated genes mediated by *AL121899.1* overexpression.

**Table S10**. Up-regulated genes in samples with *ELMO2* overexpression.

**Table S11**. Down-regulated genes in samples with *ELMO2* overexpression.
